# Pleural effusion portends a poor prognosis in patients on continuous ambulatory peritoneal dialysis

**DOI:** 10.1371/journal.pone.0297343

**Published:** 2024-01-19

**Authors:** Fengping Zhang, Ting Xiang, Xiaoran Feng, Guilin Zhang, Yu Liu, Luohua Li

**Affiliations:** 1 Department of Nephrology, Jiujiang NO.1 People’s Hospital, Jiujiang, China; 2 Department of Nephrology, Kidney Research Institute, West China Hospital of Sichuan University, Chengdu, China; 3 Department of Nephrology, The NO.1 Affiliated Hospital of Nanchang University, Nanchang, China; 4 Department of Nephrology, Pingxiang People’s Hospital, Pingxiang, China; Pikeville Medical Center, UNITED STATES

## Abstract

**Aims:**

Pleural effusion is not an infrequent complication in patients undergoing continuous ambulatory peritoneal dialysis. However, there is not adequate data to evaluate pleural effusion and prognosis in clinical practice. In this study, we validated this potential association by a multicenter cohort.

**Methods:**

We screened 1,162 patients who met the inclusion criteria with PD. According to the existence of pleural effusion on stable dialysis (4–8 weeks after dialysis initiation), the participants were divided into pleural effusion and non-pleural effusion groups. The hazard ratios (HRs) of all-cause and cause-specific death were estimated with adjustment for demographic characteristics and multiple potential clinical confounders. Subgroup analysis and propensity score matching (PSM) were used to further verify the robustness of the correlation between hydrothorax and prognosis.

**Results:**

Pleural effusion was found in 8.9% (104/1162) of PD individuals. After adjusting for the confounding factors, patients with pleural effusion had significantly increased HRs for all-cause death was 3.06 (2.36–3.96) and cardiovascular death was 3.78 (2.67–5.35) compared to those without pleural effusion. However, it was not associated with infectious and other causes of death. After PSM, the HR of all-cause mortality was 3.56 (2.28–5.56). The association trends were consistent in the subgroup sensitivity analysis.

**Conclusion:**

Pleural effusion is not rare in PD, and is significantly associated with overall and cardiovascular mortality, which is independent of underlying diseases and clinically relevant indicators.

## Introduction

Peritoneal dialysis (PD) is a form of dialysis for the survival of patients with end-stage renal disease (ESRD). Approximately 11% of dialysis patients worldwide undergo peritoneal dialysis [1, 2]. However, despite the continuous development of dialysis diagnostic technology and equipment, dialysis patients still have a poor prognosis and the incidence of health-related complications or poor quality of life remains high [3, 4]. Pleural effusion is one of the complications that cannot be ignored [5].

Although congestive heart failure and uremic toxin are the primary causes of pleural effusion in ESRD, the relevant research is still limited, especially the data on pleural effusion and mortality [6]. In recent years, several researches have mentioned that hydrothorax is an absolute risk factor for increasing mortality in chronic kidney disease (CKD) without dialysis, but in the field of dialysis, especially in PD, we did not obtain enough clinical information to assess the prognostic significance of pleural effusion [7, 8]. A complete understanding of the prognosis characteristic of patients with pleural effusion in PD may contribute to the management of this population.

## Objects and methods

### Data source

**Fig 1:**
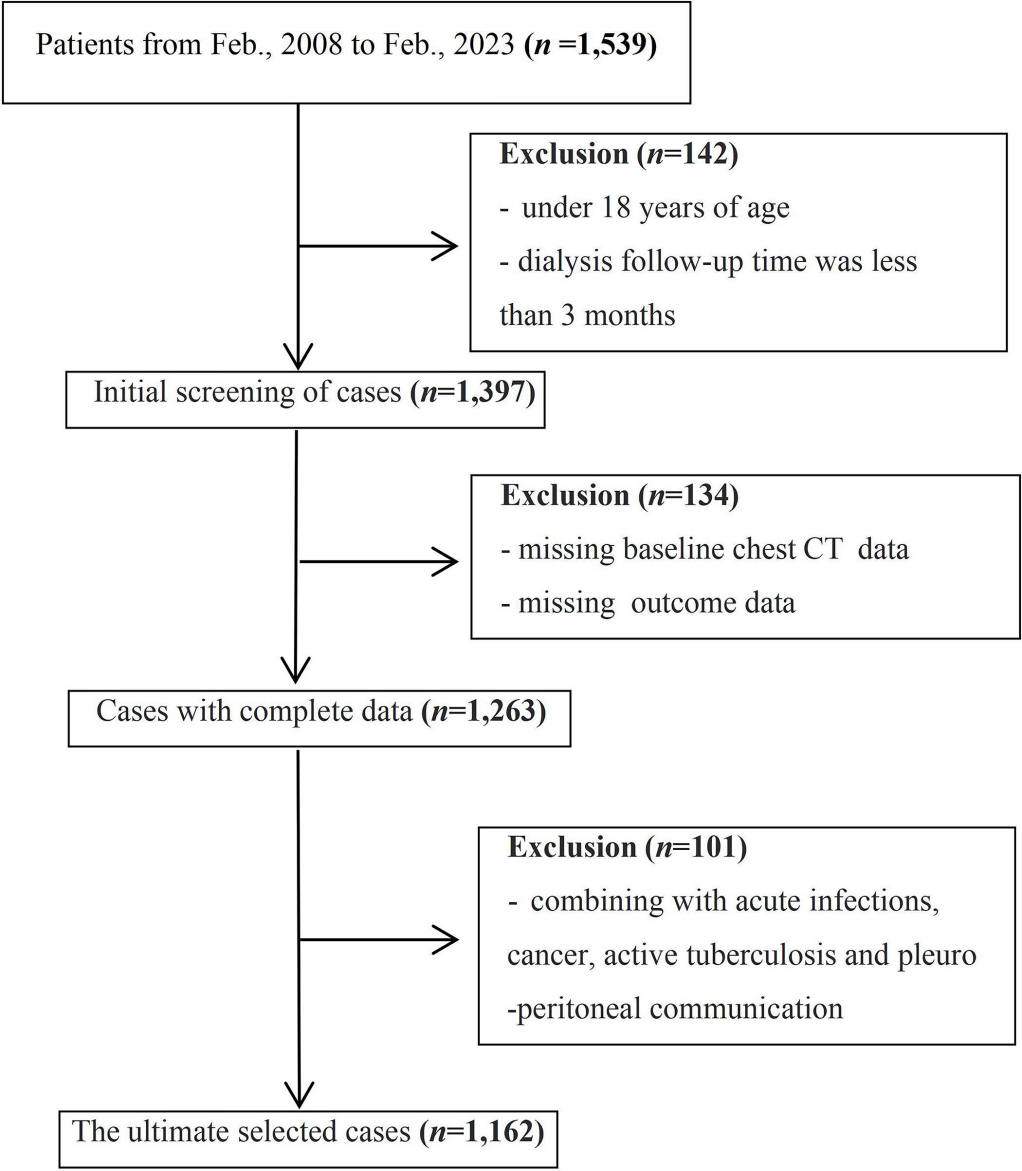
Flow-chart of the participant’s enrollment.

Our retrospective cohort study data were derived from four PD databases containing information on demographics, laboratory and imaging findings, dialysis, complications, and key clinical events in China. The design of the database was to update the input dynamically corresponding to the unified standard format, and the key information was recorded in each sub-center routinely. The study involved 1,539 initial Han ethnic adult PD population who initiated and followed up from February 2008 to February 2023 on continuous ambulatory peritoneal dialysis (CAPD: the prescription is usually 1.5% dextrose peritoneal dialysate *3 times *4 hours + 2.5% dextrose peritoneal dialysate overnight). Excluding those who were followed up for less than 3 months, lack of baseline chest imaging examination and outcome data, and acute infections (such as pneumonia and peritoneal dialysis peritonitis). Cancer, active tuberculosis and pleuro-peritoneal communication were not included in our discussion as well. The medical records of organ transplants or conversion to hemodialysis were considered as censored data. As shown in **Fig 1**, 1,162 participants were finally enrolled in our study after excluding cases that did not meet the criteria. The research unit ethics committee ratified the protocol and exempted the informed consent form based on an anonymous electronic database. We accessed the data and conducted the research after obtaining ethical approval in May 2023.

### General data and outcome

The participants’ general data includes the following aspects: 1) demographics and examination data [age, sex, diseases history, smoking, drinking, medication, body mass index (BMI), edema as assessed clinically and urine volume]; 2) clinical variables [fasting blood glucose, hemoglobin, serum albumin, blood lipids, N-terminal pro-B type natriuretic peptide (NT-proBNP), ejection fraction (EF), serum creatinine, serum calcium, serum phosphate, parathyroid hormone (PTH), C-reactive protein (CRP), residual renal function (RRF), normalized protein catabolic rate (nPCR) and chest CT]; 3) PD data: urea clearance index (Kt/*V*_ure_), peritoneal membrane transport status index (D/P_cr_). All of these baseline data were collected for analysis during dialysis 4–8 weeks of dialysis (considered to be in a stable dialysis state), and the date of chest CT imaging was used as the reference for case inclusion.

The primary outcome was all-cause death and the secondary outcome was cause-specific death, such as cardiovascular death (coronary heart disease, arrhythmia, heart failure, cerebrovascular disease, peripheral vascular disease and other related diseases), infectious death and death from other causes. Patients without death were followed until February 2023 and the endpoint event was recorded as survival.

### Determination of pleural effusion

In this study, we focused on patients with pleural effusion at the time of stable dialysis. The degree of pleural effusion was uniformly discussed and determined at each center, and the simple judgment criterion through CT mediastinal window was as follows: a) none, no obvious abnormal low-density effusion; b) mild, the medial edge of the chest wall was parallel to the pleura in an arc-shaped narrow banded liquid-like low-density shadow; c) moderate, a crescent-shaped liquid-like low-density shadow was seen on the medial edge of the posterior chest wall, and the lung tissue was slightly compressed locally; d) severe, obvious compression of lung tissue, the large liquid-like low-density shadow in the chest, and contralateral displacement of the mediastinum [9]. However, due to the particularity of ESRD patients and the inhomogeneity of follow-up individuals, not all participants’ pleural effusion properties and dynamic changes have been accurately evaluated.

### Methods and statistical analysis

Participants were divided into pleural effusion and non-pleural effusion groups based on the presence of pleural effusion during stable dialysis. All experimental data were presented by mean and standard deviation, median, and interquartile range or frequency (percentage). Fisher’s exact test, t-test, and Wilcoxon U test were used to compare the appropriate situation between the two groups. The survival rate of various categories was assessed using the Kaplan-Meier curve and log-rank test. Cox regression analysis was applied to analyze the hazard ratios (HRs) of all-cause or cause-specific death adjusted potential covariates in pleural effusion groups. The hazard times calculation began the study registration and ended with death, loss of follow-up or termination of research. To minimize the impact of potential baseline confounding factors on the association between pleural effusion and mortality, we conducted a stratified analysis of diverse variables. Additionally, we employed propensity score matching (PSM) to balance the background characteristics of the two groups, using a clamp value of 0.2. After PSM, we calculated the HRs for the cohort data. The statistical analysis was conducted using R software (version 4.3.0) and SPSS (version 26.0).

## Results

The study involved 1,162 patients, with the following characteristics: mean age, 53.6 ±14.5 years; proportion of female, 608 (52.4%); BMI, 22.0 ±3.3 kg/m^2^; cardiovascular disease, 289 (24.9%); diabetes, 264 (27.1%); connective tissue diseases, 34 (2.9%); tuberculosis history, 89 (7.6%); serum albumin, 35.0 ±4.9g/L; median RRF, 3.1 (1.8, 5.1) ml/min/1.73m^2^; total Kt/*V*_ure_, 1.76 ±0.61. There were 104 (8.9%) patients with pleural effusion [mild 45 (40.9%), moderate 34 (32.7%), severe 25 (24.0%); unilateral 80 (76.9%), bilateral 24 (23.1%)]. All participants were divided into two categories: the pleural effusion group (*n* = 104) and the non-pleural effusion group (*n* = 1,058). Statistical analysis results suggested that the age, proportion of cardiovascular disease, proportion of diabetes, NT-proBNP and CRP were higher, while BMI, serum albumin, EF and RRF were lower in the pleural effusion group. Additional parameter characteristics were available in **Table 1**. These baseline features showed that patients with pleural effusion exhibit tendencies towards advanced age, underlying diseases, malnutrition, worsened heart function, poor RRF, and inflammation.

**Table 1:** Baseline characteristics of patients with pleural effusion in all patients and PSM cohoyrt.

	All patients			PSM cohort		
	pleural effusion	non-pleural effusion	*p*-value	pleural effusion	non-pleural effusion	*p*-value
	(*n* = 104)	(*n* = 1058)		(*n* = 104)	(*n* = 104)	
Age (years)	57.6 ± 15.3	53.1 ± 14.3	0.002**	57.6 ± 15.3	56.4 ± 15.0	0.546
Female, *n* (%)	63 (60.6%)	545 (51.5%)	0.077	63 (60.6%)	61 (58.7%)	0.777
Body mass index (kg/m^2^)	21.2 ± 2.9	22.1 ± 3.3	0.006**	21.1 ± 2.9	21.8 ± 3.8	0.214
Smoking, *n* (%)	12 (11.3%)	116 (10.9%)	0.858	12 (11.3%)	11 (10.6%)	0.825
Drinking, *n* (%)	7 (6.7%)	60 (5.7%)	0.658	7 (6.4%)	9 (8.7%)	0.795
Cardiovascular disease, *n* (%)	42 (40.4%)	247 (23.3%)	<0.001**	42 (40.4%)	39 (37.5%)	0.670
Diabetes, *n* (%)	37 (35.6%)	227 (21.4%)	0.001**	37 (35.6%)	37 (35.6%)	1.000
Hypertension, *n* (%)	75 (72.1%)	799 (75.5%)	0.443	75 (70.7%)	84 (80.8%)	0.141
Connective tissue diseases, *n* (%)	6 (5.8%)	28 (2.6%)	0.071	6 (5.6%)	4 (3.8%)	0.517
Tuberculosis history, *n* (%)	10 (9.6%)	79 (7.5%)	0.432	10 (9.6%)	11 (10.6%)	0.818
Drugs, *n* (%)						
Statin	32 (30.7%)	240 (22.7%)	0.069	32 (30.7%)	38 (36.5%)	0.463
Anti-anemia	90 (86.6%)	869 (82.1%)	0.282	90 (86.6%)	88 (84.6%)	0.843
Edema, *n* (%)	11(10.6%)	93 (8.8)	0.587	11(10.6%)	9 (7.7%)	0.631
Fasting blood glucose (mmol/L)	4.9 ± 1.3	4.8 ± 1.6	0.502	4.9 ± 1.3	4.7 ± 1.4	0.170
Hemoglobin (g/L)	95.6 ± 11.2	95.9 ± 14.8	0.799	95.6 ± 11.2	95.5 ± 15.0	0.954
Albumin (g/L)	33.4 ± 5.1	35.1 ± 4.9	<0.001**	33.4 ± 5.1	34.4± 4.6	0.149
HDL-c (mmol/L)	1.2 ± 0.5	1.2 ± 0.4	0.857	1.2 ± 0.5	1.2 ± 0.5	0.963
LDL-c (mmol/L)	2.4 ± 0.9	2.5 ± 1.0	0.810	2.4 ± 0.9	2.5 ± 1.0	0.635
NT-proBNP (pg/mL)	17679 (9696, 26128)	7996 (5400, 11139)	<0.001**	17679 (9696, 26128)	15769 (12929, 21737)	0.692
Ejection fraction (%)	56.1 ± 8.6	59.6 ± 9.0	<0.001**	56.1 ± 8.6	55.4 ± 8.7	0.533
Serum creatinine (umol/L)	779 ± 239	806 ± 344	0.434	779 ± 239	767 ± 340	0.326
Calcium (mmol/L)	2.13 ± 0.28	2.12 ± 0.26	0.966	2.13 ± 0.28	2.00 ± 0.24	0.393
Phosphate (mmol/L)	2.08 ± 0.50	2.00 ± 0.57	0.166	2.08 ± 0.50	1.95 ± 0.53	0.073
Parathyroid hormone (pg/ml)	223 (114, 314)	232 (126, 370)	0.387	223 (114, 314)	221 (145, 324)	0.719
C-reactive protein (mg/L)	0.8 (0.6, 1.2)	0.6 (0.5, 0.9)	<0.001*	0.8 (0.6, 1.2)	0.8 (0.5, 1.4)	0.353
RRF (ml/min/1.73 m^2^)	2.0 (1.0, 4.5)	3.2 (1.8, 5.3)	<0.001**	2.0 (1.0, 4.5)	2.4 (1.7, 4.0)	0.213
Urine volume (ml)	640 (480, 800)	720 (560, 880)	<0.001**	640 (480, 800)	640 (550, 800)	0.568
nPCR (g/kg/day)	1.16 ± 0.23	1.21 ± 0.27	0.068	1.16 ± 0.23	1.12 ± 0.25	0.277
D/P_cr_	0.65 ± 0.13	0.68 ± 0.16	0.099	0.65 ± 0.13	0.68 ± 0.17	0.306
Total Kt/*V*_ure_	1.74 ± 0.47	1.76 ± 0.63	0.760	1.74 ± 0.47	1.71 ± 0.60	0.718

**p<0.05, ** *p*<0.01

Abbreviations: PSM, propensity score matching; HDL-c, high density lipoprotein cholesterol; LDL-c, low density lipoprotein cholesterol; NT-proBNP, N-terminal pro-B type natriuretic peptide; RRF, residual renal function; nPCR, normalized protein catabolic rate; D/P_cr,_ peritoneal membrane transport status; Kt/*V*_ure_, urea clearance index

### Pleural effusion and overall mortality

**Fig 2:**
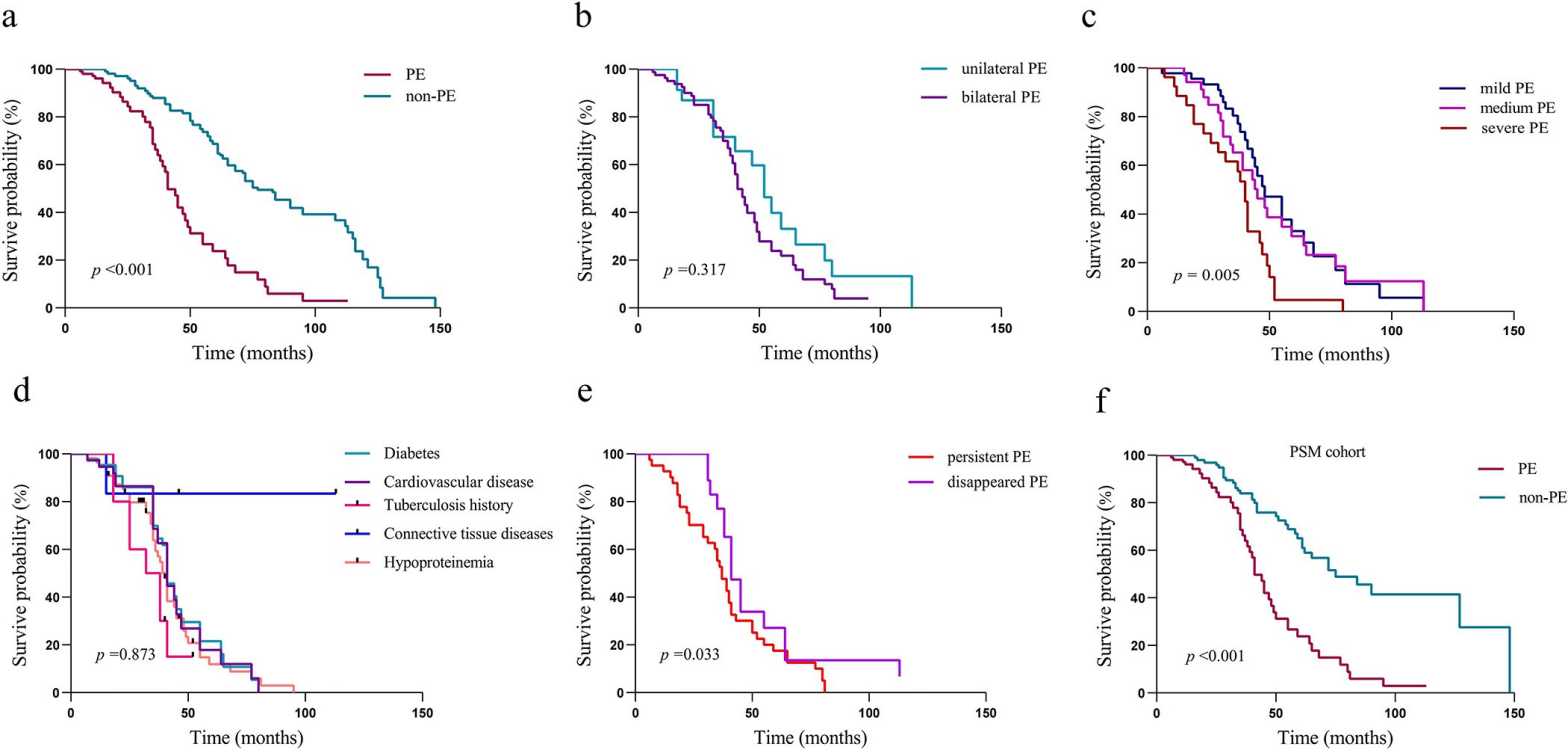
Kaplan-Meier survive curves in different pleural effusion (PE) grouping cohorts (a) All patients (b) Grouping of unilateral or bilateral in pleural effusion, (c) Grouping of severity in pleural effusion, (d) Grouping of follow-up pleural effusion, (e) Grouping of comorbidities, and (f) Patients in the propensity score matching (PSM) cohort.

During the follow-up period [median 48 (28, 63) months], 433 deaths (including 247 cardiovascular deaths, 103 infectious deaths and 83 other causes of death) were recorded. The Kaplan-Meier survival curve indicated that the overall mortality of the pleural effusion group was distinctly higher than non-pleural effusion (*p* < 0.001) (**Fig 2A**), especially in the subgroup with severe (**Fig 2B**). However, there was no correlation between the survival rate and unilateral and bilateral effusion (**Fig 2C**). After adjusting for potential confounding variables (age, sex, body mass index, comorbidity, albumin, ejection fraction, c-reactive protein, RRF, and Total Kt/*V*ure), the overall mortality in pleural effusion raised significantly with HR 3.06 (2.36–3.96) (**Table 2**). Additional results of Cox regression analyses for overall mortality can be found in **S1 Fig**.

**Table 2:** The hazard ratios of pleural effusion with all-cause and cause-specific death (refer to non-pleural effusion).

	All-cause death		Cardiovascular death		Infection death		Other causes death	
	HR (95% CI)	*p*-value	HR (95% CI)	*p*-value	HR (95% CI)	*p*-value	HR (95% CI)	*p*-value
All patients								
Unadjusted	3.53 (2.76–4.53)	<0.001**	4.06 (2.96–5.57)	<0.001**	1.89 (1.00–3.55)	0.049*	1.67 (0.94–2.97)	0.083
Adjusted model^†^	3.06 (2.36–3.96)	<0.001**	3.78 (2.67–5.35)	<0.001**	1.83 (0.93–3.58)	0.079	1.48 (0.80–2.72)	0.214
PSM cohort								
Unadjusted	3.16 (2.13–4.71)	<0.001**	3.88 (2.26–6.68)	<0.001**	1.43 (0.62–3.28)	0.398	1.48 (0.68–3.21)	0.326
Adjusted model^†^	3.56 (2.28–5.56)	<0.001**	3.66 (2.06–6.49)	<0.001**	1.20 (0.47–3.07)	0.698	1.26 (0.54–2.92)	0.592

*p<0.05

** *p*<0.01

^†^: adjusted for age, sex, body mass index, comorbidity, albumin, ejection fraction, c-reactive protein, residual renal function, and Total Kt/*V*_ure_

Furthermore, utilizing follow-up data from 62 patients with pleural effusion (where the chest CT data was taken from the event closest to the end point and laboratory data utilized mean follow-up values), it was observed that individuals with persistent pleural effusion (*n* = 41) exhibited a substantially higher mortality rate when compared to those who no longer experienced it (*n* = 21) (**Fig 2E**). The prognosis of patients with differing pleural effusion durations was significantly disparate among the 21 individuals who experienced resolved effusion. Prolonged durations of such effusion were observed to result in poorer prognoses (**S2 Fig**). Both groups displayed higher levels of NT-proBNP and CRP as well as lower EF, RRF, urine volume, and Kt/*V*ure (**Table 3**). We conducted a further Cox regression analysis using all follow-up data. The findings demonstrated that patients with persistent pleural effusion (*n* = 41) exhibited a substantially higher HR for all-cause mortality compared to those without pleural effusion (*n* = 1058) [3.26 (2.26–4.69)] (**S3 Fig**).

**Table 3:** Average follow-up data obtained from patients with pleural effusion.

	Total	Persistent group	Disappeared group	*p*-value
	(*n* = 62)	(*n* = 41)	(*n* = 21)	
Fasting blood glucose (mmol/L)	5.0 ± 1.1	4.9 ± 1.1	5.2 ± 1.2	0.287
Hemoglobin (g/L)	95.4 ± 11.0	94.6 ± 10.3	96.7 ± 12.5	0.561
Albumin (g/L)	32.7 ± 4.9	31.8 ± 4.6	34.2 ± 5.3	0.093
HDL-c (mmol/L)	1.2 ± 0.4	1.2 ± 0.5	1.3 ± 0.3	0.425
LDL-c (mmol/L)	2.5 ± 0.8	2.5 ± 0.9	2.6 ± 0.7	0.659
NT-proBNP (pg/mL)	23541(12919, 31815)	25422 (14740, 34900)	16225 (14965, 24874)	0.038*
Ejection fraction (%)	53.8 ± 7.9	52.2 ± 7.8	56.9 ± 7.2	0.024*
Serum creatinine (umol/L)	800 ± 226	796 ± 217	810 ± 247	0.819
Calcium (mmol/L)	2.11 ± 0.28	2.09 ± 0.28	2.16 ± 0.31	0.625
Phosphate (mmol/L)	1.99 ± 0.81	2.03 ± 0.75	1.89 ± 0.63	0.457
Parathyroid hormone (pg/ml)	241 (163, 336)	290 (165, 375)	188 (142, 263)	0.082
C-reactive protein (mg/L)	0.9 (0.5, 1.1)	1.0 (0.3, 1.3)	0.7 (0.5, 0.9)	0.027*
RRF (ml/min/1.73 m^2^)	1.8 (0.9, 2.7)	1.3 (0.9, 2.2)	2.7 (1.0, 3.9)	0.011*
Urine volume (ml)	410 (380, 620)	310 (210, 525)	550 (380, 770)	0.026*
nPCR (g/kg/day)	1.13 ± 0.15	1.11 ± 0.15	1.17 ± 0.15	0.157
D/P_cr_	0.59 ± 0.24	0.59 ± 0.26	0.61 ± 0.21	0.795
Total Kt/*V*_ure_	1.73 ± 0.32	1.66 ± 0.36	1.84 ± 0.30	0.040*

*: *p*<0.05; Abbreviations: HDL-c, high density lipoprotein cholesterol; LDL-c, low density lipoprotein cholesterol; NT-proBNP, N-terminal pro-B type natriuretic peptide; RRF, residual renal function; nPCR, normalized protein catabolic rate; D/P_cr,_ peritoneal membrane transport status; Kt/*V*_ure_, urea clearance index

### Pleural effusion and cause-specific mortality

According to the death classification data set, we subdivide the causes of death into cardiovascular, infection, and other causes death. The identical Cox regression model showed a similar upward trend in cardiovascular death risk for the pleural effusion group, with a HR of 3.78 (2.67–5.35). Infection-related deaths (*p* = 0.079) and other causes deaths (*p* = 0.214) did not show any significant association **(Table 2)**.

### Subgroup analysis

**Fig 3:**
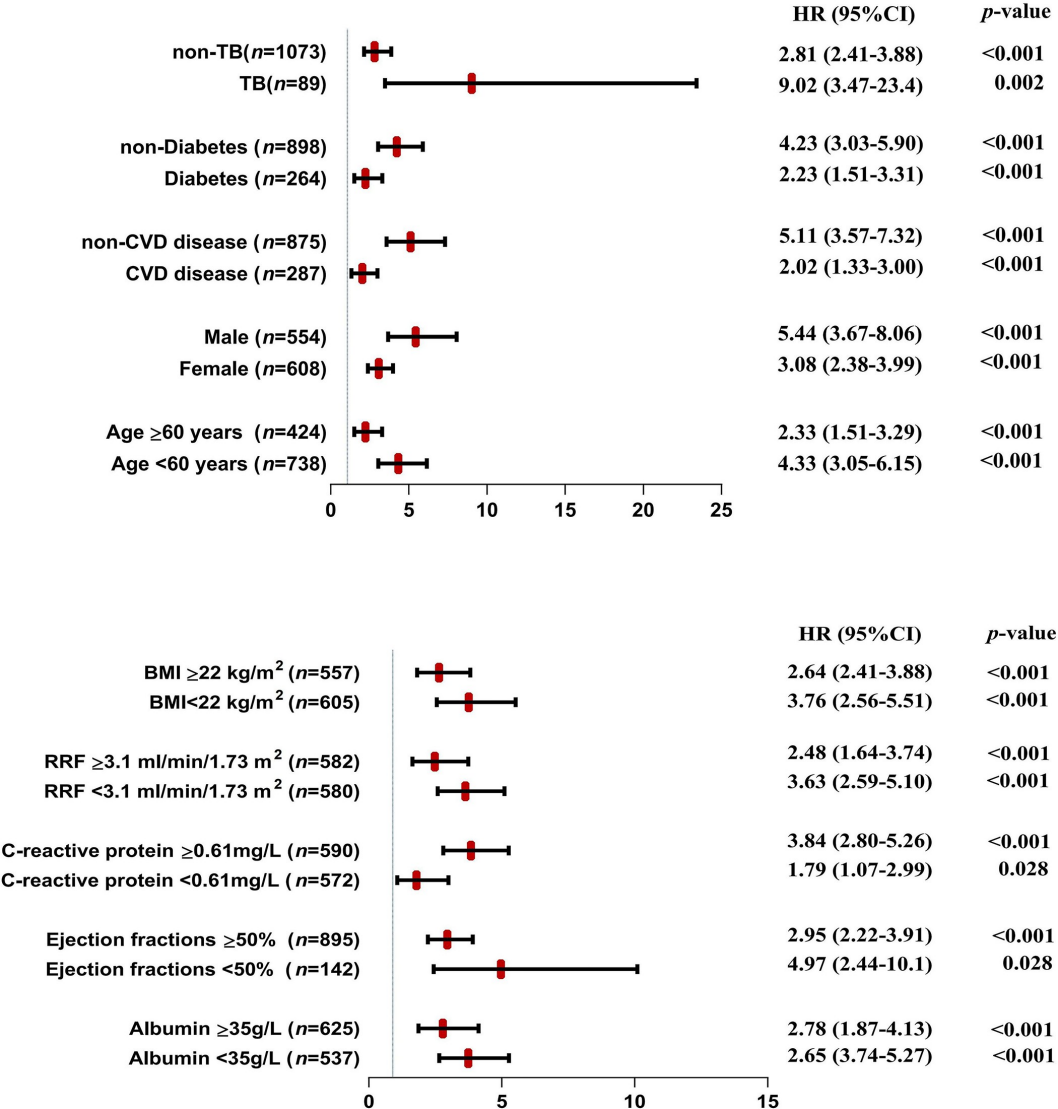
The adjusted HRs of pleural effusion and overall mortality in various subgroups of all patients.

To ascertain the mortality risk between pleural effusion groups, we calculated HRs for different subgroups such as age, sex, cardiovascular disease history, diabetes, tuberculosis history, BMI, albumin, EF, CRP, RRF, D/Pcr and Kt/*V*ure at baseline. The findings of the stratified analysis indicated that pleural effusion was related to higher mortality in all subgroups, which was essentially in line with the outcomes of the entire population. It should be noted that the hazard ratio was significantly increased in the group with a prior history of tuberculosis. (**Fig 3 and S4 Fig**).

### PSM

To balance the selection bias caused by background factors, we further matched the propensity scores of patients with pleural effusion and non-pleural effusion groups. All the differentiators, including age, BMI, disease, serum albumin, EF, CRP, and RRF were matched by propensity score, and the prognosis of the two groups was analyzed again. The standardized differences of each factor were less than 0.15 before and after matched. In the PSM cohort, the overall mortality of the group with pleural effusion was also significantly increased (**Fig 2F**). The Cox regression models also yielded similar adjusted results as before. The HRs of all-cause and cardiovascular cause of death in patients with pleural effusion were 3.56 (2.28–5.56) and 3.66 (2.06–6.49), respectively. The results showed that the mortality in patients with pleural effusion increased substantially, which was consistent with the analysis before PSM **(Table 2)**.

In short, these findings reveal a robust correlation between pleural effusions and heightened mortality in PD patients, even following adjustments for multiple variables, along with stratified analyses and propensity matching to ensure balance confounding factors.

## Discussion

Pleural effusion is a common clinical manifestation of patients with CKD 3–5 stage which affects the quality of life in a great extent [10, 11]. In this study, we found that the incidence of pleural effusion in PD patients was 8.9%, compared with 2.1% in another PD center and 31.8% of hemodialysis people reported in the past [12, 13]. The differences may stem from the evaluation methods and time point. Through the use of multivariate adjustment, stratification, and PSM sensitivity analyses, we conducted an investigation into the connection between pleural effusion and mortality, both overall and cause-specific. Our findings indicate that pleural effusion is a highly significant and consistent contributing factor. This association stands independently of the basic condition, uremic toxin, low protein, and the laterality of the effusion, but instead relates to its severity and duration. As far as we know, this is the first time to use uniform time point and assessment tools to analyze the relationship between pleural effusion and long-term mortality in patients undergoing PD on a large scale. These findings warrant medical consideration.

In general population, the presentations of pleural effusion are predominantly determined by the underlying disease, and cardiac insufficiency maybe the uppermost factor [14]. In ESRD, the emergence of pleural effusion maybe also related to uremic pleurisy, infection, and even part of the proportion of unexplained reasons [12, 15, 16]. According to the pathophysiological theory, the decrease of RRF in dialysis patients leads to fluid overload, and the accumulation of a large number of toxins inhibits the function of serous cells, platelets and other coagulation factors, resulting in increased capillary permeability of visceral and parietal pleura. Hypoalbuminemia caused by malnutrition and a decrease of colloidal osmotic pressure may be potential mechanisms of hydrothorax, and some rare cases may have to do with anatomic abnormalities such as pleural and peritoneal leakage. However, in our study, the relationship between pleural effusion and mortality was not associated with demographic characteristics, previous diseases, albumin levels, EF, RRF, and CRP, as our analysis adjusted for these confounding variables. A prospective studies as well have demonstrated that pleural effusion is independently associated with prognosis and is a marker of advanced disease [17].

In the initial data set, it’s explicit that pleural effusion is susceptible to the senior, previous complications, hypoproteinemia, worse heart function, poor RRF and inflammation. In fact, these factors are recognized as a potent marker of inferior prognostic [18, 19]. However, in the PSM analysis adjusted for background factors and sensitivity analysis, we strongly observed the independent relevance between hydrothorax and overall mortality and cardiovascular mortality. Interestingly, the statistical results also showed that the HR of cardiovascular mortality was slightly higher than all-cause mortality, and not associated with infection. Especially in the persistent pleural effusion group, the NT-proBNP and EF were considerably different from the population in which the effusion was relieved. Clinical studies have confirmed that NT-proBNP and EF are valuable indicators of circulatory load, lung congestion, cardiac function, and are closely related to the prognosis of dialysis [20, 21]. We speculate that the negative effect of hydrothorax on prognosis may be related to exposure of cardiovascular disease due to added capacity load in the future. Meanwhile, pleural effusion may contribute to additional protein loss, leading to malnutrition-inflammation and atherosclerosis (MIA) syndrome, thus affecting the prognosis, these characteristics can be disclosed in Tables 2 and 3. Nevertheless, due to the incomplete follow-up data, we assume that the specific mechanism of poor prognosis caused by pleural effusion needs to be confirmed by deeper studies.

For nephrologists, the removal of hydrothorax is a meaningful issue in clinical activities. Shorter duration of effusion was associated with better survival, which corroborates the benefit and importance of early resolution of the effusion. It has been reported that strengthening dialysis, puncture and drainage and the use of broad-spectrum antibiotics may be beneficial to the alleviating of pleural effusion [12, 22, 23]. However, mechanical removal may be a challenge for dialysis patients and is unknown for long-term improvement, and whether broad-spectrum antibiotics are avail in non-infection-related effusion. In addition, for a rare but serious type of pleural effusion—pleuro-peritoneal communication, only termination of overtreatment of CAPD to IPD, or suspension of peritoneal dialysis to overtreatment of hemodialysis and fistula repair may help to improve the effusion of such patients. As we all know, dialysis adequacy affects the volume load and overall survival rate of dialysis patients [24]. Inadequate dialysis may lead to adverse situations such as fluid overload and accumulation of uremic toxins. Our data showed that there is no significant difference in Kt/*V*_ure_ and D/P_cr_ between two groups at baseline, suggesting that the pre-existing state of effusion is not due to poor dialysis itself, and the influence of dialysis-related indicators on long-term prognosis was not attenuated. The most common treatments for patients with pleural effusion by doctors in our dialysis center were improving dialysis adequacy, infusion of albumin, increasing diuresis, and transient automated peritoneal dialysis (APD) or thoracentesis. Interestingly, we found that during follow-up after stable dialysis, the Kt/*V*_ure_ and overall survival rate of patients with the disappearance of effusion status were superior to the persistent, which suggests that raising the adequate performance of dialysis may be conducive to lighten excessive systemic volume load and toxin accumulation in PD patients. Even more to the point, we should interpret this information carefully, on the grounds that we cannot determine the causality and obtain more complete data to explain the findings at a deeper level.

Our study has distinct advantages. Firstly, we employed multiple centers, large sample sizes, and unified time cut-off points to identify hydrothorax, which reduces potential diagnostic deviations caused by differences in dialysis duration. Secondly, we balanced confounding factors such as pleural effusion and risk of death by adjusting various clinical factors and employing PSM, which enhances the validity of our findings. Furthermore, this study excluded parapneumonic effusions resulting from acute pneumonia. These types of pleural effusions can generally be resolved rapidly with enhanced anti-infection measures, and are not commonly associated with long-term prognosis. In addition, laboratory indicators such as CRP, serum albumin, and Kt/*V*ure are not consistently useful in cases of acute infection and must be considered accordingly.

This article focuses on the prognostic impact of pleural effusion at baseline on dialysis, which may introduce some limitations in this study. Firstly, we obtained little detailed data on the nature of the pleural effusion. As the patients are in a state of dialysis, most of them do not undergo pleural puncture, which makes it impossible for us to determine the cause of the effusion. We failed to confirm whether transudates or exudates make a difference in prognosis. Some apparently heterogeneous causes of pleural effusion, such as acute pulmonary tuberculosis and cancer are not included in this discussion, although these cases were uncommon in our cohort. Secondly, the selection bias caused by retrospective cases is an inevitable disadvantage, which may miss some cases since we diagnosed pleural effusion relying on CT alone. Finally, the complete dynamic changes of effusion are not in our discussion. Partial patients may have no pleural effusion during the baseline period, but emerge at a later stage. Therefore, further prospective studies are still needed to demonstrate these results.

## Conclusion

In conclusion, our finding indicated that the existence of pleural effusion has a significant negative effect on prognosis and is independently related to higher mortality in PD.

## Supporting information

S1 Fig The results of Cox regression analyses with overall mortality in all patients (*n* = 1,162). Abbreviations: PE, pleural effusion; *β*,β values for the cox analysis; *SE*, standard error.
(JPG)

S2 Fig Kaplan-Meier survive curves between different duration of effusion in the patients (*n* = 21) whose pleural effusion (PE) disappeared. (JPG)

S3 Fig The results of Cox regression analyses with overall mortality in patients with persistent pleural effusion (*n* = 41) and non-pleural effusion (*n* = 1,058). Abbreviations: PE, pleural effusion; *β*,β values for the cox analysis; *SE*, standard error.
(JPG)

S4 Fig The adjusted HRs of pleural effusion and overall mortality in various subgroups of all patients (D/Pcr and Kt/*V*ure). (JPG)
