# Supplementary figures and images for: Pleural effusion portends a poor prognosis in patients on continuous ambulatory peritoneal dialysis
Source: PLoS One. 2024 Jan 19;19(1):e0297343. doi: 10.1371/journal.pone.0297343 (PMC10798541; doi:10.1371/journal.pone.0297343)

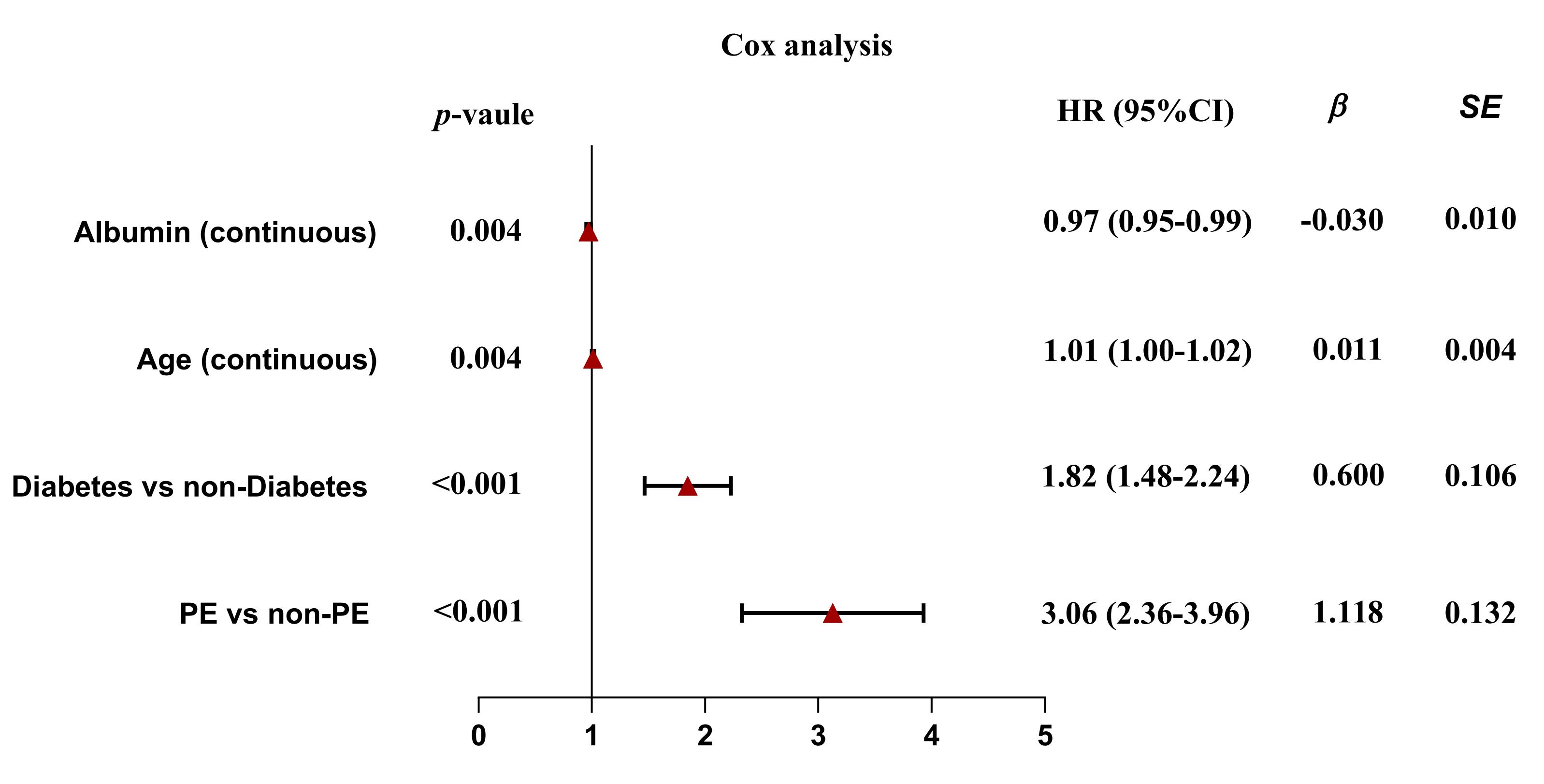

Supplement: S1 Fig — Abbreviations: PE, pleural effusion; β,β values for the cox analysis; SE, standard error. (JPG) [file pone.0297343.s001.jpg]

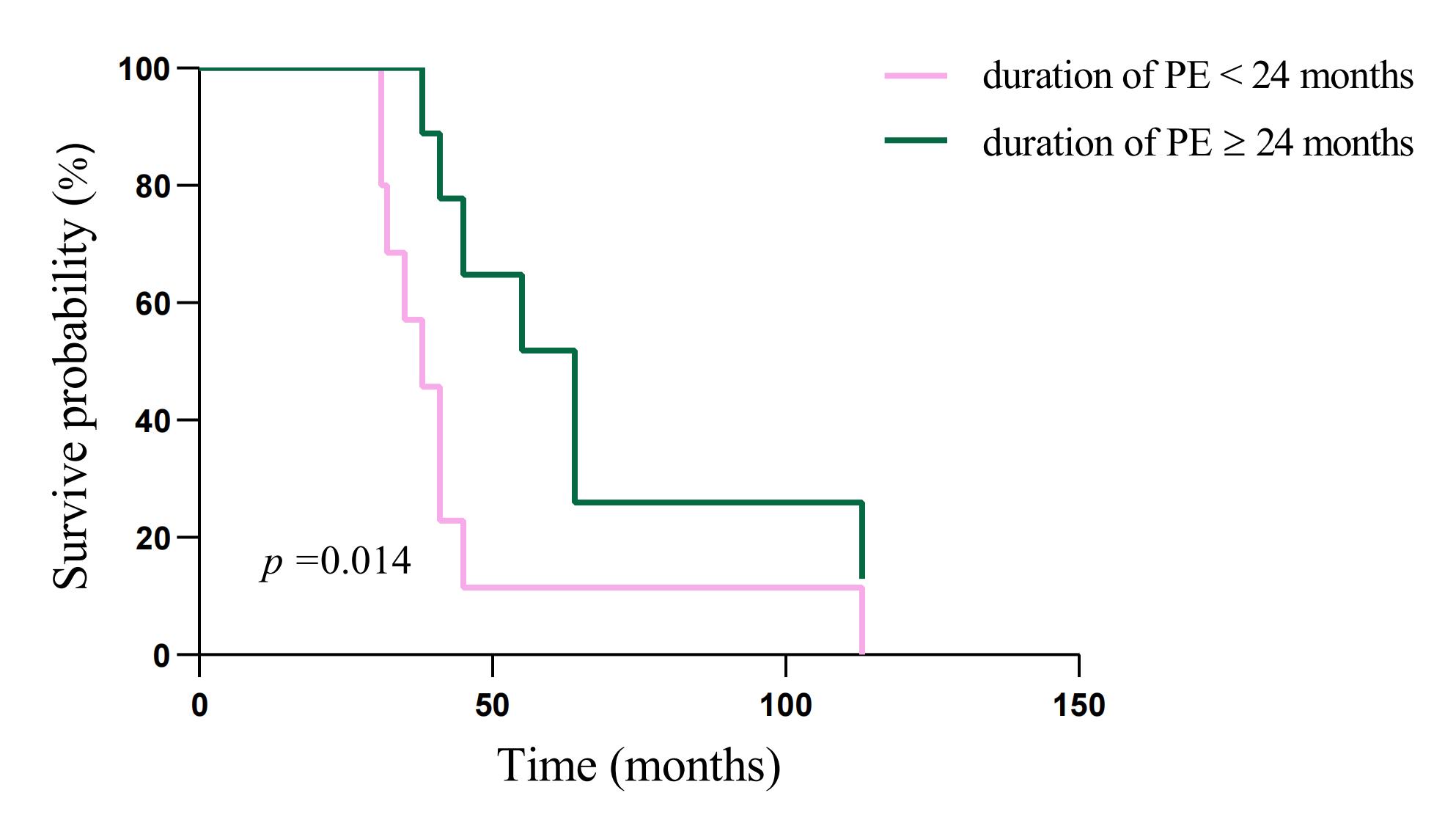

Supplement: S2 Fig — (JPG) [file pone.0297343.s002.jpg]

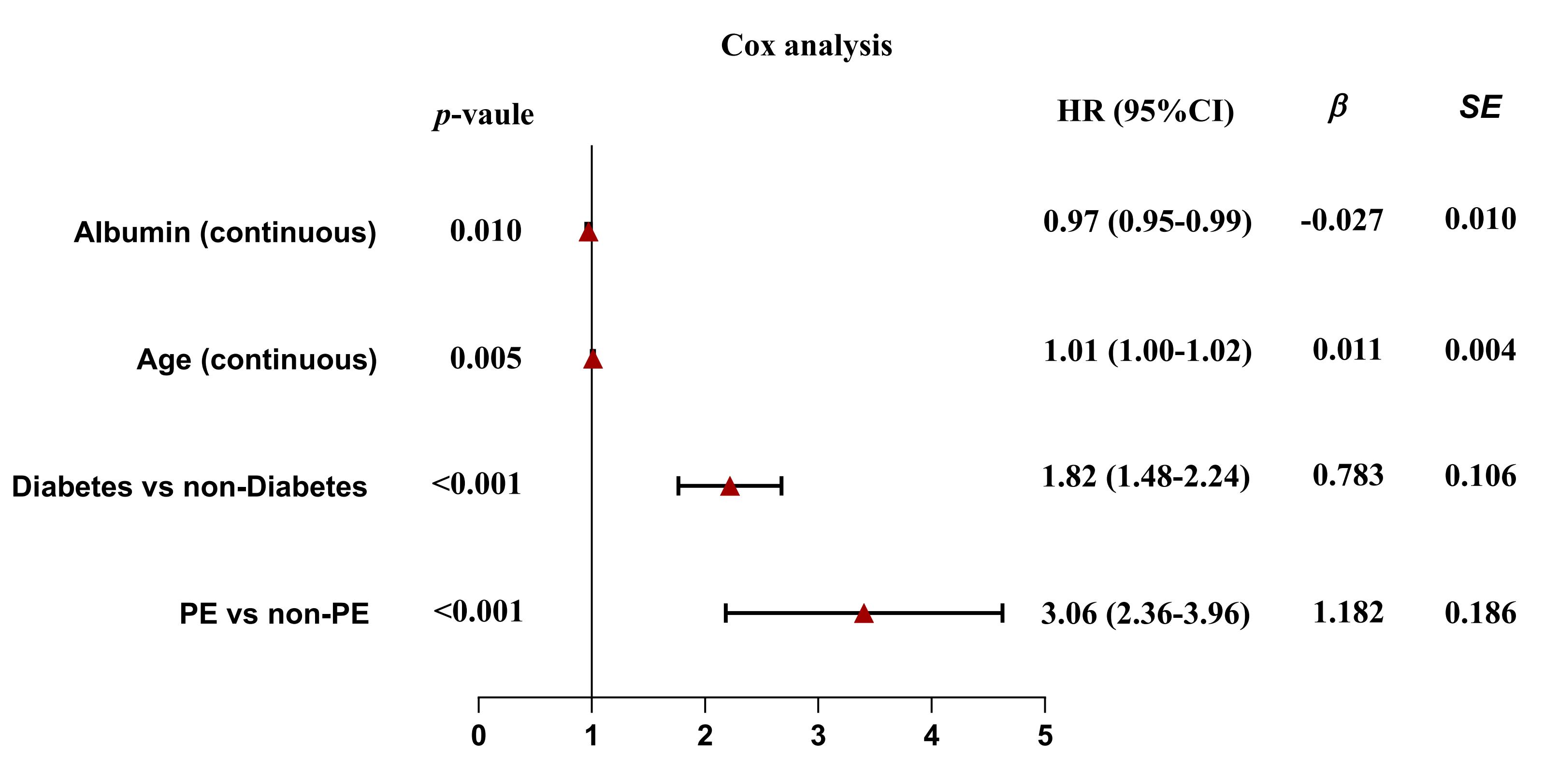

Supplement: S3 Fig — Abbreviations: PE, pleural effusion; β,β values for the cox analysis; SE, standard error. (JPG) [file pone.0297343.s003.jpg]

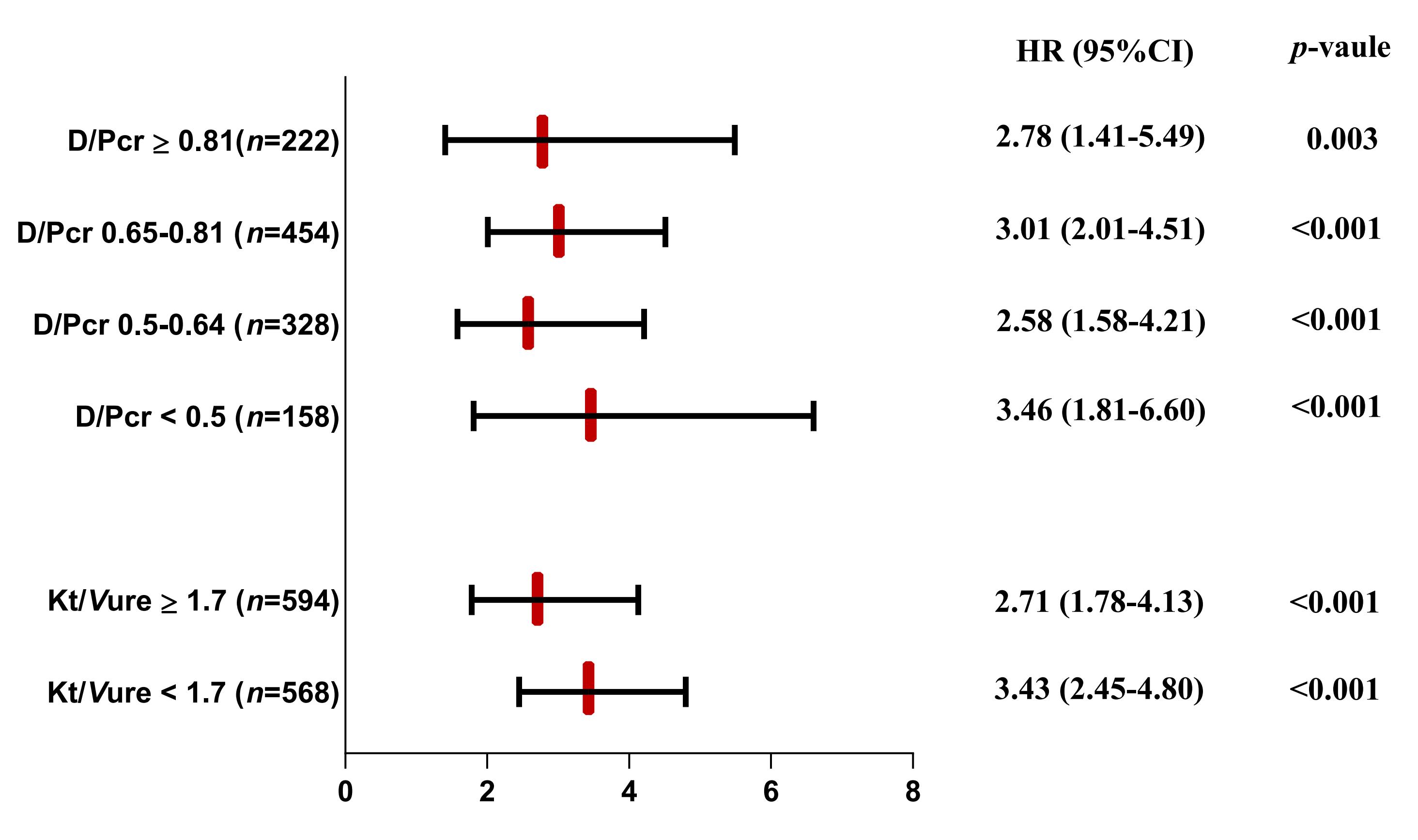

Supplement: S4 Fig — (JPG) [file pone.0297343.s004.jpg]
